# Verapamil Inhibits *Aspergillus* Biofilm, but Antagonizes Voriconazole

**DOI:** 10.3390/jof3030050

**Published:** 2017-09-20

**Authors:** Hasan Nazik, Varun Choudhary, David A. Stevens

**Affiliations:** 1 California Institute for Medical Research, 2260 Clove Dr., San Jose, CA 95128, USA; varun746@gmail.com (V.C.); stevens@stanford.edu (D.A.S.); 2 Division of Infectious Diseases and Geographic Medicine, Stanford University Medical School, Stanford, CA 94305, USA

**Keywords:** verapamil, *Aspergillus*, biofilm, voriconazole

## Abstract

The paucity of effective antifungals against *Aspergillus* and increasing resistance, the recognition of the importance of *Aspergillus* biofilm in several clinical settings, and reports of verapamil—a calcium channel blocker—efficacy against *Candida* biofilm and hyphal growth, and synergy with an azole antifungal in vitro, led to a study of verapamil ± voriconazole against *Aspergillus*. Broth macrodilution methodology was utilized for MIC (minimum inhibitory concentration) and MFC (minimum fungicidal concentration) determination. The metabolic effects (assessed by XTT [2,3-bis[2-methoxy-4-nitro-5-sulfophenyl]-2H-tetrazolium-5-carboxanilide inner salt]) on biofilm formation by conidia were studied upon exposure to verapamil, verapamil plus voriconazole, or voriconazole alone. For biofilm formation, we found less inhibition from the combinations than with either drug alone, or less inhibition from the combination than that of the more potent drug alone. For preformed biofilm, we found no significant change in activity comparing voriconazole alone compared to added verapamil, and no significant alteration of activity of the more potent voriconazole, at any concentration in the range tested, by addition of a concentration of verapamil that is inhibitory alone. In full checkerboard assays with planktonic fungus, there was no indication of any effect of one drug on the other (indifference). Although verapamil was similarly inactive against planktonic *Aspergillus*, as with *Candida*, verapamil was indeed active against *Aspergillus* biofilm. However, indifference and antagonism was found with voriconazole.

## 1. Introduction

In eukaryotes, the calcium signaling pathway plays an essential role in response to cellular stress, including oxidative stress. On perception of stress by the cell, there is a calcium influx, leading to the upregulation of calcium-dependent genes that are required for the response to stress. Verapamil is a calcium channel blocker. Its effect is to decrease calcium fluctuation and thus the calcium influx, decreasing the oxidative stress response.

In *Candida albicans*, the depression of the calcium flux by verapamil results in increased sensitivity to oxidative stress, increased reactive oxygen species, and mitochondrial dysfunction, with a net result of fungal inhibition [1]. In addition, many fungi maintain a cytoplasmic calcium gradient, with high calcium concentrations at the growing tip, a necessary condition for polarized growth [2]. Thus in *C. albicans*, the calcium signaling pathway is necessary for morphogenetic transformation to the hyphal state, the more invasive state of the organism. Blockage of the calcium flux by verapamil inhibits hyphal development [3]. In addition, candidal adherence and the ability to colonize the mammalian gut are decreased by verapamil treatment. Calcium homeostasis is also necessary for biofilm development [4]. Verapamil has activity in vitro against the formation and maintenance of candidal biofilm, and acts synergistically with antifungals against candidal biofilm formation [5].

Although a number of effective antifungal drugs have been developed against *C. albicans*, the antifungal armamentarium against *Aspergillus* is more limited [6], and resistance development to the agent of choice—voriconazole [7]—is increasing [8]. *Aspergillus* biofilm formation is a problem in several clinical situations, particularly chronic lung infections, such as in cystic fibrosis [9,10]. Verapamil has a well-studied pharmacology and toxicology [11]. We therefore investigated whether verapamil, alone or in combination, could offer something promising against *Aspergillus* biofilm.

## 2. Materials and Methods

For reagents, Verapamil was obtained from Hospira (Lake Forest, IL, USA) and Sigma (St. Louis, MO, USA) and compared in the assays of inhibition to be described. The potency from these two sources was found to be equivalent, in saline or in RPMI1640 medium, and they were then used interchangeably. We also noted no differences if verapamil stocks were first constituted in distilled water or dimethyl sulfoxide (DMSO). Voriconazole was obtained from Pfizer, New York City, and stock was made in DMSO for further dilution down to test conditions with RPMI. A control of the same DMSO concentration without voriconazole verified no effect of DMSO alone. Large batches of the reagents were prepared in aliquots and frozen, and a fresh aliquot used in each experiment.

For *Aspergillus* biofilm inhibition assays, *Aspergillus fumigatus* (strain AF10, a virulent clinical isolate) (ATCC^®^ 90240™) [12] biofilm formation and preformed biofilm were prepared as previously detailed [13]. The presence of biofilm was verified: hyphal mats with biofilm form [9] were visualized by optical microscopy, showing the same arrangement as has been noted in optical, confocal and electron microscopy [13,14]. A 96-well plate assay [15] was used, with RPMI1640 medium, 8 replicate wells/each experimental group, preparation of the Af target from conidiating cultures (on potato dextrose agar) (PDA), and XTT (2,3-bis[2-methoxy-4-nitro-5-sulfophenyl]-2H-tetrazolium-5-carboxanilide inner salt) (Sigma) readout of Af inhibition, as previously described [13,15]. In brief, for studies of biofilm formation, 2 × 10^3^ conidia/well was co-cultured with reagents for 16 h at 37 °C, shaking 65–70 RPM. At 16 h, a biofilm had formed, as noted previously [13]. For study of preformed *Aspergillus* biofilm, reagents were added only after 16 h, then incubated for 24 h. After 40 h, the metabolic activity of the biofilm was measured with XTT assay. XTT results of each experimental group were compared to those of a concurrent *Aspergillus* control (no added reagents), by *t*-test. Biofilm formation was studied in six experiments, and preformed biofilm in nine experiments.

For MIC and MFC determination, two additional Af clinical isolates, CIMR nos. 15–31 and 15–37 were also studied. Broth macrodilution methodology was utilized as described [16], including concurrent testing of a known voriconazole-susceptible isolate as a control for drug batch activity. Minimum fungicidal concentrations (MFCs) were determined by subculture of the drug-treated tubes, with an MFC endpoint of ≥96% killing of original inoculum [17].

Data from experiments using XTT were analyzed using one-way ANOVA with a Tukey’s post-test for multiple comparisons and Student’s *t*-test was used if two groups were compared. All statistical analyses were performed using Graph-Pad Prism (GraphPad Software, version 3, La Jolla, CA, USA). Statistical significance was considered *p* < 0.05.

## 3. Results

**Figure 1:**
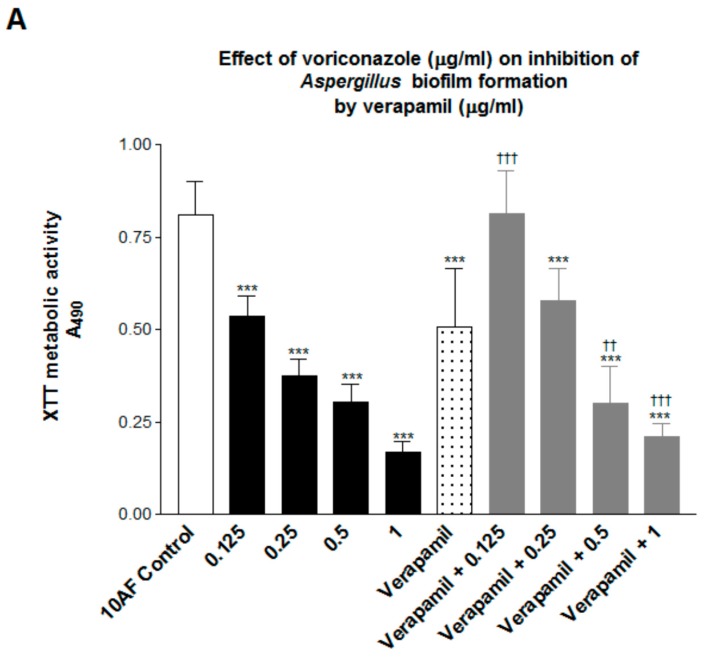

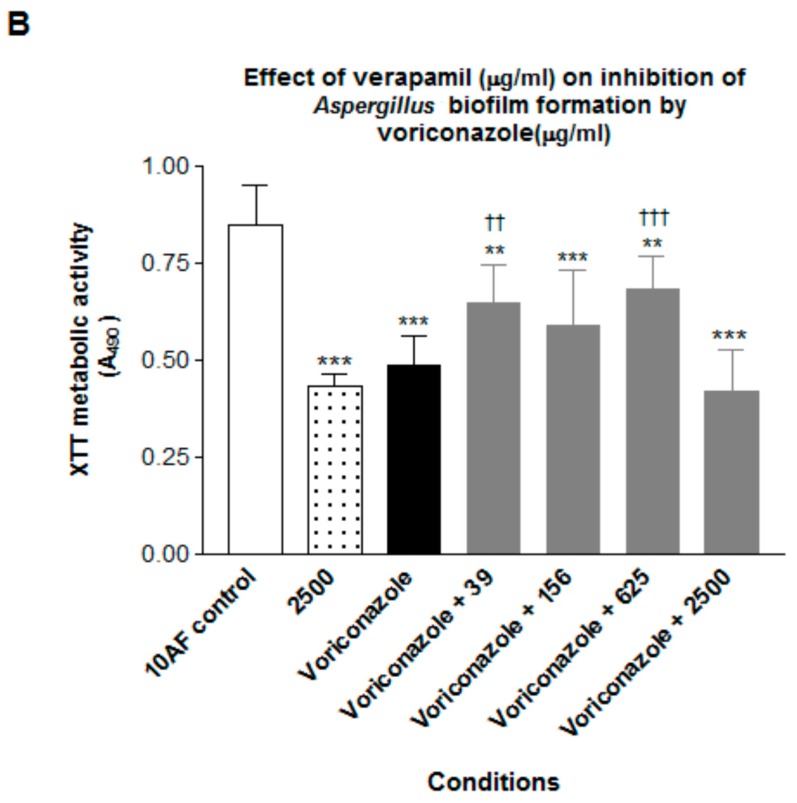
(**A**) Verapamil 39 mcg/mL alone was inhibitory, as shown here (sixth bar from left). This is a study of the effect of voriconazole doses on the effect of verapamil on formation of *Aspergillus* biofilm. The numbers 0.125 to 1 are final voriconazole concentrations in mcg/mL, alone (second through fifth bars from the left) or in combination with verapamil 39 mcg/mL (four right bars). ***, *p* < 0.001 lower than the no drug control, left bar. ^††^ and ^†††^ are *p* < 0.01 and 0.001, respectively, vs. verapamil alone. Verapamil + 0.125 voriconazole is not only less inhibitory than verapamil alone (^†††^), but also than 0.125 voriconazole alone (*p* < 0.001). Verapamil + 0.25 voriconazole is not only less inhibitory than verapamil alone, but also less inhibitory than 0.25 voriconazole alone (*p* < 0.01). Verapamil + 1 mcg/mL voriconazole is not only less inhibitory than verapamil alone (^†††^), but also less inhibitory than 1 mcg/mL voriconazole alone (*p* < 0.05). (**B**) The effect of verapamil doses on the effect of voriconazole on formation of *Aspergillus* biofilm can be seen. The numbers 39 to 2500 represent the final concentration of verapamil, alone or in combination, in mcg/mL. The final voriconazole concentration, alone or in combination was 0.125 mcg/mL. ** and ***, *p* < 0.01 and 0.001, respectively, compared to no drug control (left bar). ^††^ and ^†††^, *p* < 0.01 and 0.001, respectively, less inhibition in combinations compared to voriconazole alone. Antagonism is again demonstrated.

**Figure 2:**
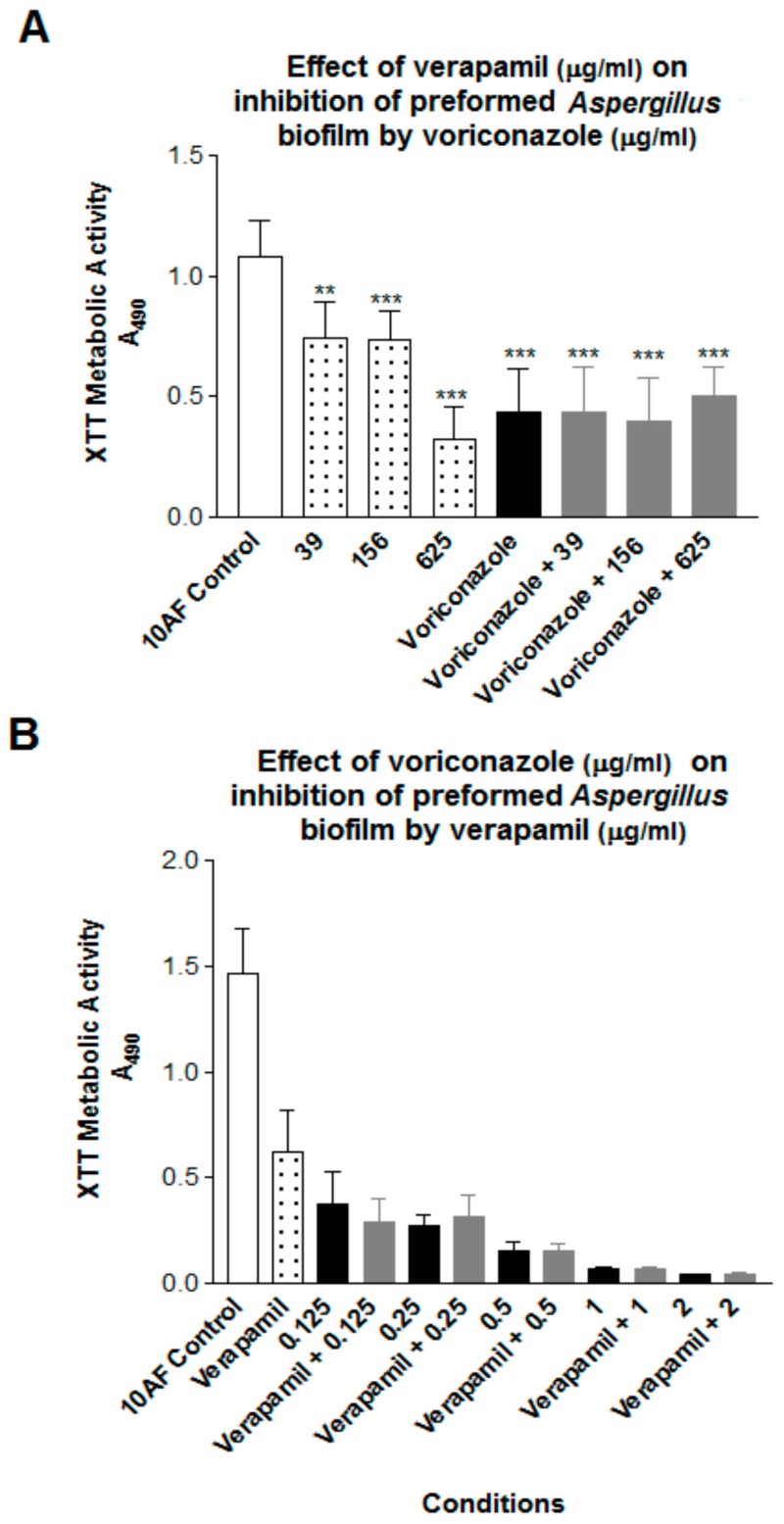
(**A**) Effect of verapamil doses on the effect of voriconazole on preformed Af biofilm. The voriconazole dose tested was 0.125 mcg/mL final concentration (4 right bars). The numbers 39, 156, 625 refer to verapamil final concentrations in mcg/mL. Two and three asterisks refer to *p* < 0.01 and 0.001, respectively, compared to no-drug control (left bar). The combination bars are not different from voriconazole alone; no potentiation of voriconazole by verapamil. (**B**) Effect of voriconazole doses on the effect of verapamil on preformed Af biofilm. The verapamil concentration tested, alone (second bar from left) or in combination, was 39 mcg/mL. The numbers 0.125 through 2 are final concentrations of voriconazole in mcg/mL, alone or in combination. The no-drug control is at the left, and is *p* < 0.001 different from all other bars. The voriconazole alone bars are plotted here side-by-side with the corresponding combination bars; there are no differences in any pair. Indifference is again demonstrated.

The initial studies were performed to examine the dose response for both reagents, in two- and four-fold dilution ranges. For biofilm formation, concentrations of verapamil 2500 to 39 mcg/mL showed significant (*p* < 0.01–0.001) inhibition compared to the no drug control. The lowest concentration of verapamil that gave repeated statistically significant inhibitory results were 7.8 mcg/mL for preformed biofilm, and 39 mcg/mL for assays of biofilm formation. For voriconazole, a range of 0.125–2 mcg/mL gave consistent inhibition for both preformed biofilm and biofilm formation, and this was satisfactory for our purposes, i.e., to provide a range from which concentrations could be selected for combination studies, allowing sufficient ‘room’ to demonstrate in the combination tubes either enhancement (additive or synergy response) or loss of activity (antagonism) compared to the single drug tube. For studies of drug interactions, it was desirable to use a concentration of one that gave inhibition compared to the control in the mid-range—i.e., approximately 50%—and then examine the effect of a dilution series of the other drug. This is because the laboriousness, and time needed, in preparing the wells with biofilms and executing the experiments precludes doing a full checkerboard assay concurrently (as is possible in MIC assays). Thus it is desirable to have a drug level of the test drugs singly in the mid-range so as to be able to see, and test statistically, greater inhibition in the combination (indicating an additive effect or synergy) than one drug alone (i.e., a value below that of the one drug alone), or less inhibition (indicating antagonism, a value of the combination above that of the one drug alone), if either is present. We tested several concentrations of the single drugs in these assays to give us these desired conditions. Representative experiments are shown in Figure 1 and Figure 2.

In Figure 1 (biofilm formation) we see the less inhibition by the combinations than that with either drug alone, or less inhibition from the combination than that of the more potent drug alone. These are defined as antagonism.

For preformed biofilm a dose-response for verapamil alone is shown in Figure 2. In Figure 2 (preformed biofilm) we see no significant change in activity comparing voriconazole alone vs. added verapamil (Figure 2A), and no significant alteration of activity of the more potent voriconazole, at any concentration in the range tested, by addition of a concentration of verapamil that is inhibitory alone (Figure 2B), both results shown in Figure 2 defined as indifference.

A full checkerboard assay was performed using three strains in planktonic growth (data not illustrated). A range of voriconazole susceptibilities was included. In these studies the MIC of voriconazole alone was 0.5, 0.25, and 0.125 mcg/mL for strains 10AF, 15–77 and 15–31, respectively, if a 50% inhibition endpoint was used for the MIC, and 4, 1, and 1 mcg/mL, respectively, if a 100% endpoint (clear tube) was used. The MFCs were >8, 1 and 1 mcg/mL, respectively. The MIC and MFC of verapamil was >624 mcg/mL with all isolates and either endpoint. There was no indication of any effect of one drug on the other (indifference).

## 4. Discussion

The lack of verapamil activity against *A. fumigatus* planktonic growth is consistent with the report of inactivity against *C. albicans* planktonic growth (inactivity at 320 mcg/mL, different medium) [1]. The concentrations in our study that inhibited *Aspergillus* biofilm formation approximate those reported to inhibit *C. albicans* biofilm formation (20 mcg/mL) [5], a concentration that also inhibited the metabolism of preformed *C. albicans* biofilm, and approximated what we found with preformed *Aspergillus* biofilm.

There were some unexpected findings from our studies. First, we could not show the synergy of verapamil with an azole against *Aspergillus* biofilm, either its formation or on preformed biofilm—as has, in contrast, been reported with an azole and *C. albicans* biofilm [5]—and our interaction was in part a negative one. The reason for those differences is a subject for future research. It will be important in such studies to examine the applicability of our findings to other, clinical, *A. fumigatus* isolates, and for our findings to be confirmed by other investigators.

Second, although it has been generally held that *Aspergillus* biofilm is resistant to voriconazole [18,19,20], that was not our finding. Those differences could relate to differences in medium used, differences in *A. fumigatus* strains, and/or differences in assay methods, and endpoint determinations. It has also been reported [18] that preformed *Aspergillus* biofilm is more drug-resistant than biofilm formation, whereas we did not find this to be the case with either drug studied, although the reference cited was not addressing a drug such as verapamil, our verapamil concentrations inhibitory to biofilm formation and preformed biofilm were not distantly disparate, and our voriconazole concentrations were not titered below 0.125 mcg/mL (which then possibly could have shown differences).

The relevant serum concentrations of verapamil in its cardiologic use are 0.1–0.4 mcg/mL [11]. This would suggest that verapamil itself would not be promising for therapy either against planktonic *A. fumigatus* or *C. albicans*, their biofilms, or in combination with azoles, given the verapamil endpoints derived in our studies. Verapamil is also an inhibitor of the p-glycoprotein drug efflux pump [21], a property that could also affect these drug combination studies against fungi, but this property might also affect penetration of other (e.g., antifungal) drugs into tissues. However, our findings and these cautions do not preclude the possible utility of other calcium channel blockers as adjuncts to antifungal therapy, particularly against biofilm homeostasis.
